# Lactate-upregulation of lactate oxidation complex-related genes is blunted in left ventricle of myocardial infarcted rats

**DOI:** 10.1590/1414-431X20187660

**Published:** 2018-10-04

**Authors:** D. Gabriel-Costa, T.F. Cunha, N.A. Paixão, R.S. Fortunato, I.C.C. Rego-Monteiro, M.L.M. Barreto-Chaves, P.C. Brum

**Affiliations:** 1 Programa de Pós-Graduação em Desempenho Humano Operacional, Universidade da Força Aérea, Rio de Janeiro, RJ, Brasil; 2 Departamento de Biodinâmica do Movimento do Corpo Humano, Escola de Educação Física e Esporte, Universidade de São Paulo, São Paulo, SP, Brasil; 3 Instituto de Biofísica Carlos Chagas Filho, Universidade Federal do Rio de Janeiro, Rio de Janeiro, RJ, Brasil; 4 Departamento de Anatomia, Instituto de Ciências Biomédicas, Universidade de São Paulo, São Paulo, SP, Brasil

**Keywords:** Lactate, Lactate oxidation complex, Myocardial infarction, Gene expression, Perfusion pressure, Oxidative stress

## Abstract

Lactate modulates the expression of lactate oxidation complex (LOC)-related genes and cardiac blood flow under physiological conditions, but its modulatory role remains to be elucidated regarding pathological cardiac stress. The present study evaluated the effect of lactate on LOC-related genes expression and hemodynamics of hearts submitted to myocardial infarction (MI). Four weeks after MI or sham operation, isolated hearts of male Wistar rats were perfused for 60 min with Na^+^-lactate (20 mM). As expected, MI reduced cardiac contractility and relaxation with no changes in perfusion. The impaired cardiac hemodynamics were associated with increased reactive oxygen species (ROS) levels (Sham: 19.3±0.5 *vs* MI: 23.8±0.3 µM), NADPH oxidase (NOX) activity (Sham: 42.2±1.3 *vs* MI: 60.5±1.5 nmol·h^−1^·mg^−1^) and monocarboxylate transporter 1 (*mct1*) mRNA levels (Sham: 1.0±0.06 *vs* MI: 1.7±0.2 a.u.), but no changes in superoxide dismutase (SOD), catalase, NADH oxidase (NADox), and xanthine oxidase activities. Lactate perfusion in MI hearts had no additional effect on ROS levels, NADox, and NOX activity, however, it partially reduced *mct1* mRNA expression (MI-Lactate 1.3±0.08 a.u.). Interestingly, lactate significantly decreased SOD (MI-Lactate: 54.5±4.2 µmol·mg^−1^·min^−1^) and catalase (MI: 1.1±0.1 nmol·mg^−1^·min^−1^) activities in MI. Collectively, our data suggest that under pathological stress, lactate lacks its ability to modulate the expression of cardiac LOC-related genes and the perfused pressure in hearts submitted to chronic MI. Together, these data contribute to elucidate the mechanisms involved in the pathogenesis of heart failure induced by MI.

## Introduction

Lactate is a metabolic intermediary compound that links energy metabolism to different organs and tissues (1). The heart is an important lactate consumer especially during physiological stress conditions such as exercise (2–4). We and others have demonstrated that lactate modulates the expression of lactate oxidation complex (LOC)-related genes in both cardiac and skeletal muscle cells, which drive its usage as substrate fuel (5,6). Lactate modulation of LOC-related genes occurs through the activation of redox-sensitive signaling pathways since lactate increases reactive oxygen species (ROS) levels and nuclear expression of the nuclear factor erythroid 2-related factor 2 (NRF-2), a redox-sensitive transcription factor (5,6). In fact, lactate modulates redox homeostasis in cardiac tissue by increasing the levels of reduced β-nicotinamide adenine dinucleotide (NADH) mainly due to the conversion of lactate to pyruvate by lactate dehydrogenase (LDH), which further activates a specific oxidase (NADH oxidase, NADox) leading to ROS production. Lactate also has an essential role in cardiac blood perfusion since it induces coronary artery relaxation. Montoya et al. (7) showed that it caused vasodilation in isolated aortic rings and coronary arteries in a nitric oxide (NO)-dependent manner. We have also observed that lactate perfusion reduces cardiac perfusion pressure in healthy isolated hearts (5).

Under pathological stress conditions, lactate exerts beneficial functions in energy synthesis. In heart failure (HF), both metabolic shifts of cardiac metabolism toward carbohydrate utilization and cardiac increase of lactate transport are adaptations that improve cardiac function, especially after myocardial ischemia (8). Many studies have demonstrated that oxidation of carbohydrates and other glycolytic substrates improve cardiac performance efficiency, resulting in enhanced heart contractility and less O_2_ utilization (8,9). Taking into consideration data from healthy hearts, which demonstrate that lactate up-regulates the expression of LOC-related genes and increases coronary artery blood flux (5), we can hypothesize that it may also influence the establishment of HF. However, to our knowledge, no other study has previously investigated this hypothesis. The up-regulation of LOC-related gene expression and coronary artery vasodilation in hearts submitted to acute and chronic ischemia can be important compensatory mechanisms to prevent the decrease of cardiac performance and rescue the heart from further additional ischemic events, maybe delaying the onset of HF.

Considering the high indexes of mortality and the incidence of HF worldwide, especially from ischemic etiology (10,11), it is extremely relevant to investigate the mechanisms related to the establishment of the disease, increasing the knowledge about its pathogenesis and further contributing to the development of new treatment strategies. Therefore, the aim of the present work was to investigate the effect of lactate perfusion in isolated hearts submitted to chronic experimental MI. We hypothesized that lactate would increase LOC-related gene expression and decrease cardiac perfusion pressure as observed previously in non-ischemic hearts (5).

## Material and Methods

### Animals

Male Wistar rats weighing 250–300 g were used in the present study. Rats were placed in acrylic boxes lined with wood chips and access to water and standard chow *ad libitum*. Experiments were conducted according to the procedures stated in the Guide for the Care and Use of Laboratory Animals (National Institutes of Health) and approved by the Ethics and Research Committee of the Universidade de São Paulo (#2011/55).

### Myocardial infarction surgery

MI was obtained by ligation of the anterior descending coronary artery (ADCA). Animals were anesthetized with ketamine (50 mg/kg, *ip*) and xylazine (10 mg/kg, *ip*) and after thoracotomy, the ADCA was occluded preventing left ventricle (mainly anterolateral wall) blood supply. Some animals underwent the same procedures except the coronary artery ligation and were included in the sham-operated group. The day after surgery, echocardiographic analysis was conducted, and only infarcted animals that had lack of motility of the left ventricle anterior wall in M mode were included in the study. The animals were then assigned to three experimental groups: sham-operated perfused with Krebs-Henseleit (KH) solution (Sham), MI perfused with KH solution (MI), and MI perfused with KH solution plus sodium lactate (20 mM) (MI-lactate).

### Isolated heart preparation

After four weeks of the MI or sham surgery, the animals were euthanized and their hearts removed and placed on a Langendorff apparatus to test the effect of lactate perfusion as previously described by our group (5). Briefly, the isolated hearts were attached to a metal cannula in the Langendorff apparatus through the aorta for retrograde reperfusion. The flow was continuously kept throughout the experiment (±9 mL/min), and the hearts of Sham and MI groups were perfused with KH solution of composition in: 118.0 mM NaCl; 4.7 mM KCl; 1.66 mM MgSO_4_; 1.18 mM KH_2_PO_4_; 1.5 mM CaCl_2_; 24.88 mM NaHCO_3_; 2.0 mM glucose. The hearts of the MI-lactate group were perfused with the same solution containing 20 mM sodium lactate (KH plus lactate=KHL). During the perfusion, the solutions were filtrated (Swinnex filter holder: 47mm, membrane pore: 0.22 μm; EMD Millipore, USA), bubbled with a carbogenic mixture (95% CO_2_ and 5% O_2_) and maintained at 37°C and pH±7.4. Subsequently, the left ventricle was accessed through the mitral valve, and a needle puncture was used to perforate the heart apex to avoid liquid accumulation. After that, a soft latex distensible balloon was placed into the left ventricle and inflated until diastolic pressure attained ±10 mmHg. Both aortic and balloon cannulas were connected to pressure transducers and signals were amplified, digitalized, and stored for further analysis of the developed pressure (DP), heart rate (HR), maximum positive and negative dP/dt (+dP/dt_max_ and −dP/dt_max_) and perfusion pressure (PP) (Power Lab-Lab Chart 7, ADInstruments, USA).

### Experimental protocol

After the placement of the latex balloon into left ventricle, the hearts were equilibrated for 40 min beating spontaneously. KH or KHL were perfused for 60 min as previously described by Gabriel-Costa et al. (5). After 60 min, the left ventricle was dissected and readily frozen in liquid nitrogen and then at –80°C for further analysis.

### Determination of lactate in cardiac homogenate and perfusate

The lactate levels in the cardiac tissue and perfusate (collected at 0, 30, and 60 min) were evaluated based on the technique of Rosenberg and Rush (12). Briefly, the tissues or perfusates were treated with perchloric acid (3%, v/v), homogenized and centrifuged at 10,000 *g* for 20 min at 4°C. The supernatant was used to measure lactate concentration. To determine lactate levels, the samples were first incubated with 0.2 M of glycine-semicarbazide buffer and 0.02 M of NAD^+^, and the absorbance was measured at a wavelength of 340 nm (R1). Then, 2 mg/mL of LDH, obtained from rabbit muscle, was added in the preparation and incubated for 60 min at 40°C. After that, a second reading was collected at the same wavelength (R2). R1 and R2 were used to calculate the net absorbance with and without LDH, with the following formula A = (R2 – 0.9R1) – (B2 – 0.9B1), where A is the net absorbance, B1 is the blank absorbance without LDH, and B2 is the blank absorbance with LDH. The concentration of lactate in mM was inferred by using the values of the net absorbance in a standard curve obtained between absorbance and increasing concentrations of lactate.

### Determination of NOX and NADox activities and reactive oxygen species levels

NADox and nicotinamide adenine dinucleotide phosphate oxidase (NOX) activities were measured in enriched microsomal membrane (EMM) and enriched plasma membrane (EPM) of the perfused hearts, respectively. The EMM was obtained by homogenization of left ventricle tissue with phosphate buffer containing: 50.0 mM sodium phosphate, 0.5 mM dithiothreitol, 1.0 mM ethylene glycol-bis(2-aminoethyl ether)-N,N,N0,N0-tetraacetic acid, 250.0 mM sucrose, pH 7.2, and 5 μg/mL aprotinin, 34.8 μg/mL phenylmethanesulfonylfluoride (PMSF). The homogenate was centrifuged at 3,000 *g* for 15 min at 4°C. The supernatant obtained was centrifuged for 30 min at 4°C at 100,000 *g* and the pellet resuspended in 1 mL of the same buffer. Another ultracentrifugation was obtained at the same conditions, and finally, the supernatant was resuspended in 1 mL of a phosphate buffer of composition: 50.0 mM sodium phosphate, 1.0 mM EGTA, 2.0 mM MgCl_2_, 250.0 mM sucrose, and 5 μg/mL aprotinin and 34.8 μg/mL PMSF, pH 7.2. The EPM was obtained by centrifugation of homogenized left ventricle tissue at 3,000 *g* for 15 min at 4°C and another subsequent centrifugation to obtain the pellet of the preparation (13).

Both NADox and NOX were measured under similar conditions. The samples were incubated in a medium containing phosphate buffer (150 mM), pH 7.2, Amplex Red (50 μM), superoxide dismutase (SOD) (100 U/mL), and horseradish-peroxidase (0.5 U/mL). The reactions occurred with or without the corresponding substrates NADH or NADPH (0.1 mM), respectively. To calculate the specific activity of both enzymes, the slope of the curve with the addition of NADH/NADPH was subtracted from the slope of the curves with addition of water for each sample. The fluorescence was detected at a wavelength of 563 nm to excitation and 587 to emission. A standard curve with known concentrations of H_2_O_2_ was used to transform the values to nmol or µmol H_2_O_2_·min^-1^·mg^-1^. Since the preparations contained SOD, the results were referred as total H_2_O_2_ (5).

ROS generation was measured in EMM after reperfusion since NADox is present in microsomal fractions. The conditions used in this assay were similar to those employed in enzymatic measurements. The samples were incubated only with 50 μM of Amplex Red, 100 U/mL of SOD, and 0.5 U/mL of horseradish-peroxidase (HRP) for 35 min at 30°C. After that, fluorescence levels at the end of the curve (Plato) were used to infer the ROS generation using a formula obtained in a standard curve with known concentrations of H_2_O_2_ and fluorescence transforming the values in μM of H_2_O_2_.

### Quantification of xanthine oxidase activity

Xanthine oxidase (XO) activity was obtained as previously described by Veskoukis et al. (14). Samples were incubated with sodium potassium phosphate (33 mM, pH 7.5) and xanthine (0.17 mM) and the reaction was immediately stopped with trichloroacetic acid. The samples were then centrifuged at 10,000 *g* for 15 min at 4°C and absorbance was read at 293 nm. Subsequently, the same procedure was conducted after the incubation of the samples for 20 min at 37°C. XO activity was obtained by subtracting the second from the first absorbance values. Calculation of XO activity was based on molar extinction coefficient of uric acid.

### Quantification of SOD and catalase activities

SOD activity assay was based on the inhibition of xanthine/XO-driven cytochrome C reduction by SOD present in the sample. Left ventricle homogenates were obtained by macerating the tissue with potassium phosphate buffer containing: 50.0 mM KH_2_PO_4_, 50.0 mM K_2_HPO_4_, pH 7.8, followed by centrifugation at 10,000 *g* for 20 min at 4°C. The rate of cytochrome C reduction inhibition was measured in the absence or in the presence of the sample in a reaction medium containing: 1.18 mM xanthine, 19.0 mM cytochrome C, and XO, diluted in sodium phosphate buffer (50 mM, pH 7.8) for 5 min. SOD activity was calculated subtracting the rate of cytochrome C oxidase reduction inhibition in the presence and absence of the samples (15).

Catalase activity was measured as described by Weydert and Cullen (16). Muscle homogenates were obtained as described above. The rate of H_2_O_2_ decomposition by catalase was assessed by following the decay in absorbance at 240 nm for 4 min in the presence of 10 mM H_2_O_2_.

### RNA extraction and quantitative real-time RT-PCR

Total RNA was isolated from left ventricle samples using Trizol (Invitrogen, USA). The RNA concentration and purity (260:280 nm ratios) were assessed in a spectrophotometer (Nanodrop 2000, Thermo Scientific, USA) and integrity was observed in an agarose gel (2%, w/v) electrophoresis. The cDNA was synthesized using Revertaid First Strand cDNA synthesis kit (Fermentas, USA). The genes analyzed were: *nrf-2*, monocarboxylate transporter (*mct*1), 4 (*mct4*), *ldh*, peroxisome proliferator receptor coactivator type 1 alpha (*pgc1-α*), and cytochrome oxidase IV (*coxiv*) and cyclophilin were used as a reference genes. All primers were synthesized by Fermentas and their sequences are shown in Table 1. The amplifications were obtained using Maxima SYBR Green/ROX qPCR Master Mix (Fermentas) in ABI Prism 5700 Sequence Detection System (Applied Biosystems Inc., USA). Results are reported using the comparative cycle threshold (Ct) method as described by the manufacturer. The ΔΔCt calculated from the subtraction of the ΔCt of the gene of the MI and MI-lactate groups (ΔCt was calculated by subtraction of the gene Ct's from the reference-cyclophilin Ct) from the ΔCt of the control group (Sham) are reported in 2^-ΔΔCt^. Control group levels were arbitrarily set to 1.

**Table 1:** qRT-PCR primer sequences.

Gene	Forward	Reverse
*nrf-2*	5′ GGCAGGAGCTATTTTCCATTCCCGAG 3′	5′ CTGGGGACAGTGGTAGTCTCAGCCTGC 3′
*mct1*	5′ ACCGAGAGGGTCAGTGTTTG 3′	5′ TGGAGGTAAGACTGCGTCAA 3′
*mct4*	5′ GGTCCCCTGGCTGCTATTAT 3′	5′ TCCCATGGTCACACAAAGAA 3′
*ldh*	5′ GCAGCAGGGTTTCTATGGAG 3′	5′ TGGAGACAGTGGGATTGTCA 3′
*pgc1-α*	5′ GCGGACAGAACTGAGAGACC 3′	5′ CCATCATCCCGCAGATTTAC 3′
*coxiv*	5′ GAACAAGGGCACCAATGAGT 3′	5′ GTTGACCTTCATGTCCAGCA 3′
Cyclophilin	5′ TGGCAAGCATGTGGTCTTTGGGAAG 3′	5′ GGTGATCTTCTTGCTGGTCTTGCCATTC 3′

*nrf2*: Nuclear factor erythroid-2 related factor 2; *mct1*: monocarboxylate transporter 1; *mct4*: monocarboxylate transporter 4; *ldh*: lactate dehydrogenase; *pgc1-α*: peroxisome proliferator receptor coactivator type 1 alpha; *coxiv*: cytochrome oxidase IV.

### Statistical analysis

Data are reported as means±SE. The means were compared with non-paired *t*-test or one-way analysis of variance and Tukey's *post hoc*, when necessary. The results were considered significantly different when P<0.05.

## Results

Lactate levels in the perfusate were obtained at 30 min and 60 min. No differences were observed between Sham and MI groups (2.1±0.2 *vs* 1.8±0.1 mM, respectively) at 60 min, but it increased significantly after lactate perfusion (MI-lactate group: 22.3±0.8 mM). These values were kept constant throughout the experiment (30, 60, 90 min; data not shown). Cardiac lactate basal levels were not altered by MI (1.0±0.9 *vs* 1.5±1.0 mM, for Sham and MI groups, respectively). However, lactate perfusion induced a significant increase in lactate levels when MI-lactate hearts were compared to both Sham and MI hearts (4.7±0.8 mM). We also tested whether lactate administration would acidify the isolated heart perfusate along the 60 min of lactate perfusion at 20 mM (Table 2). As expected, there were no sign of acidosis in the perfusate throughout the experiment.

**Table 2:** pH values of perfusate during 60 min of perfusion with KH or KHL.

Cardiac perfusate	0 min	30 min	60 min
Sham	7.39±0.1	7.41±0.3	7.45±0.1
MI	7.4±0.2	7.5±0.1	7.51±0.1
MI-lactate	7.45±0.1	7.45±0.2	7.5±0.2

KH: Krebs-Henseleit; KHL: Krebs-Henseleit+lactate (20 mM); MI: myocardial infarction. Data are reported as means±SE of 6 hearts.

**Figure 1:**
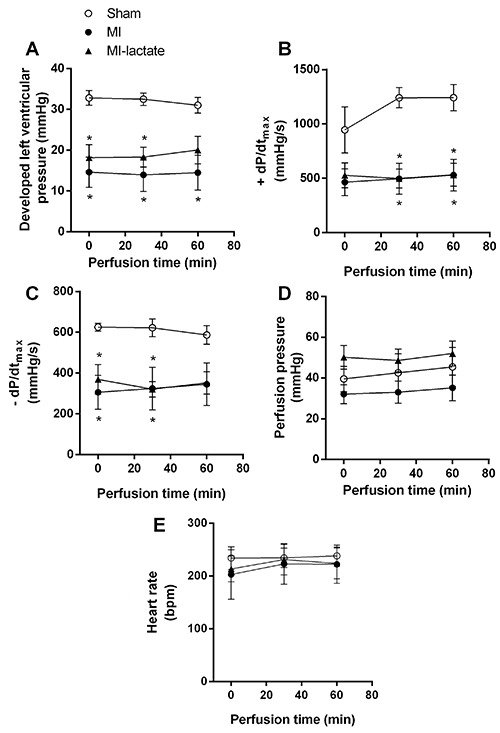
*A*, Left ventricular developed pressure, *B*, maximum positive (+dP/dt_max_ and *C*, negative (-dP/dt_max_), *D*, perfusion pressure, and *E*, heart rate after 60 min of perfusion with Krebs-Henseleit or KH plus lactate. MI: myocardial infarction. Data are reported as means±SE of 6 hearts. *P<0.05 compared to Sham group (ANOVA).

The cardiac hemodynamic parameters recorded during 60 min of perfusion of the hearts with KH or KHL solutions are shown in Figure 1. As expected, MI significantly reduced DP (Figure 1A), +dP/dt_max_ (Figure 1B), and −dP/dt_max_ (Figure 1C), and lactate perfusion had no impact on cardiac contractile function. Surprisingly, lactate perfusion did not change PP in the MI-lactate group (Figure 1D). Additionally, HR (Figure 1E) was not altered by either lactate perfusion or MI.

**Figure 2:**
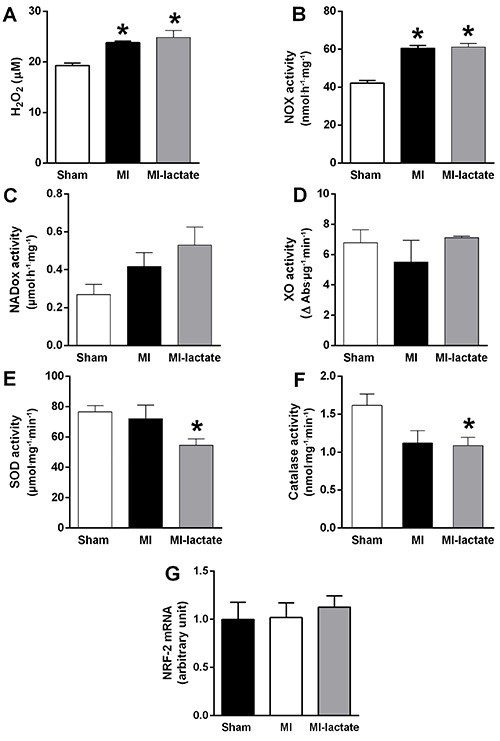
*A*, Reactive oxygen species (O_2_˙^-^/H_2_O_2_) and activity of *B*, nicotinamide adenine dinucleotide phosphate oxidase (NOX), *C*, NADH oxidase (NADox), *D*, xanthine oxidase (XO), *E*, superoxide dismutase (SOD), *F*, catalase, and *G*, total nuclear factor erythroid 2-related factor 2 (*nrf-2*) mRNA levels in hearts perfused for 60 min with Krebs-Henseleit (KH) and KH plus lactate. MI: myocardial infarction. Data are reported as means±SE of 6 hearts. *P<0.05 *vs* Sham group (ANOVA).

Figure 2A shows that MI induced a significant increase in cardiac ROS levels, which was not modified by lactate perfusion (19.8±0.4 *vs* 23.8±0.27 and 24.3±1.3 µM, for Sham, MI, and MI-lactate groups, respectively). The increased cardiac ROS levels in both MI groups were associated to a high NOX activity in MI groups (Figure 2B, 42.5±1.3 *vs* 60.5±1.5 and 61.1±1.8 nmol·h^-1^·mg^-1^, for Sham, MI, and MI-lactate groups, respectively), while NADox (Figure 2C) and XO (Figure 2D) activities remained similar among groups. Likewise, both SOD (Figure 2E) and catalase (Figure 2F) activities were not altered in MI hearts. Unexpectedly, the activity of both antioxidant enzymes was reduced by lactate perfusion in MI hearts (SOD: 76.5±4.1 *vs* 72.0±9.0 and 54.5±4.2 µmol·mg^−1^·min^−1^ and catalase: 1.61±0.15 *vs* 1.12±0.16 and 1.08±0.10 nmol·mg^−1^·min^−1^, for Sham, MI, and MI-lactate groups, respectively). The reduced SOD and catalase activities in lactate-perfused MI hearts were not accompanied by changes in *nrf-2* mRNA levels, a redox-sensitive transcription factor that regulates SOD and catalase expression (Figure 2G).

**Figure 3:**
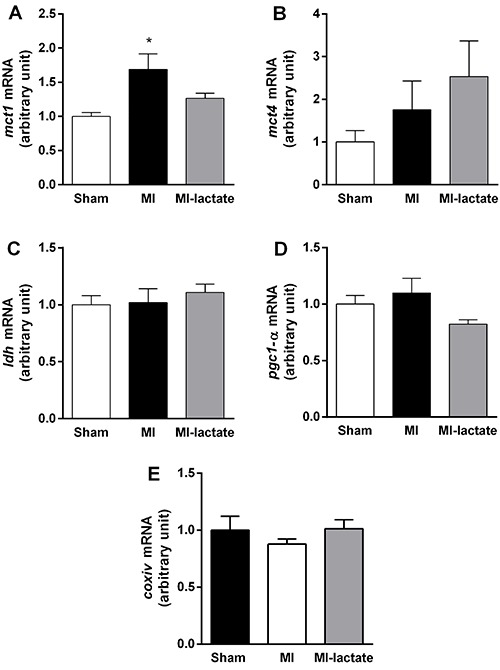
*A*, Total mRNA levels of monocarboxylate transporter (*mct*)1, *B*, *mct4*, *C*, lactate dehydrogenase (*ldh*), and *D*, proliferator receptor coactivator type 1 alpha (*pgc1-α*) and *E*, cytochrome oxidase IV (*coxiv*) in left ventricle tissue after 60 min of perfusion with Krebs-Henseleit (KH) and KH plus lactate. MI: myocardial infarction. Data are reported as means±SE of 6 hearts. *P<0.05 *vs* Sham group (ANOVA).

As lactate transportation through cell and mitochondria membranes in cardiac cells is mainly mediated by *mct1* and *mct4*, we have evaluated *mct1* and *mct4* mRNA levels in isolated hearts. *mct1* mRNA levels were significantly increased after MI, however, lactate perfusion reduced *mct1* mRNA levels toward Sham group levels (Figure 3A, 1.0±0.05, 1.6±0.22, and 1.2±0.07 a.u for Sham, MI, and MI-lactate, respectively). *mct4* mRNA levels were not modified by either MI or MI-lactate perfusion (Figure 3B). Likewise, no changes were observed in mRNA levels of other LOC-related genes among groups, such as *ldh* (Figure 3C), *pgc1-α* (Figure 3D), and *coxiv* (Figure 3E).

## Discussion

The main finding of the present study is that lactate lacks its modulatory role in the expression of LOC-related genes and in the relaxation of coronary arteries in hearts submitted to chronic pathological stress, such as MI. In parallel, the infarcted hearts presented a sustained increase in ROS levels, and no changes in *mct4*, *ldh, pgc1-α*, and *coxiv* mRNA levels were observed in either MI or lactate-perfused MI groups. In contrast, the levels of *mct1* mRNA were partially reduced, and SOD and catalase activities decreased after lactate perfusion in MI isolated hearts.

We have previously demonstrated in healthy isolated hearts that lactate induced a slight, but significant increase in ROS production that was mainly associated with NADox activity, *nrf-2* nuclear expression, and LOC gene expression (5). Presently, we demonstrated that this response was lacking in MI hearts, since we observed that MI *per se* increased ROS levels (20 µM) in cardiac cells with no further increment after lactate perfusion. In fact, the enhancement of ROS concentration was associated to NOX but not NADox activity. These data are in agreement with an established consensus, which states that MI induces both sympathetic nervous and renin-angiotensin systems hyperactivity that recognizably up-regulate components of NOX complex and ROS levels in cardiac muscle (17–19). The lack of response to lactate perfusion after MI suggests that an excessive pro-oxidant environment induced mainly by NOX activation somehow prevented the ability of lactate in modulating gene expression by redox-sensitive signaling pathways.

The lactate-induced pro-oxidant status resulted in responses other than up-regulation of LOC-related genes. In fact, lactate partially reduced *mct1* expression. Although the mechanisms involved in these effects were not investigated herein, we consider these findings relevant to HF treatment, as they contribute to elucidate the role of lactate in the establishment of the disease. Based on previous data of our group, we hypothesized that after chronic ischemia, lactate could contribute to enhancing energy metabolism by increasing the expression of proteins involved in its oxidation. It is reasonable to suggest that up-regulation of LOC-related gene expression is a positive adaptation that may further contribute to delay HF onset. However, the present data do not support this hypothesis. Other interesting data obtained were that lactate perfusion in MI hearts decreased both catalase and SOD activities. From our knowledge, this is the first study suggesting that lactate negatively modulates antioxidant enzyme activities in MI hearts. The physiological consequences of reduced antioxidant defense in HF are deleterious since oxidative stress is directly involved in its pathogenesis. Therefore, the results of the present study suggest that lactate might be one of the factors that contribute to the pathogenesis of HF.

As expected, MI-induced cardiac dysfunction was demonstrated by reduced cardiac DP, and impaired cardiac inotropic (+dP/dt) and lusitropic (−dP/dt) function, which were not changed by lactate perfusion in isolated MI hearts. Interestingly, lactate perfusion in MI hearts did not reduce cardiac perfusion pressure as we previously observed in isolated healthy hearts (5). We believe that it was in consequence of the MI-induced endothelium dysfunction, since Montoya et al. (7) have previously demonstrated that lactate-induced vasodilation was endothelium-NO-dependent. Data obtained from lactate and pH levels, both in perfusate and cardiac tissue, suggest that the hearts were well-oxygenated and relying on aerobic metabolism. Our data corroborate the results of Opie (20), who used similar perfusion pressures.

Taken together, our data provide evidence that MI blunted the ability of lactate in modulating LOC-related gene expression and cardiac perfusion in isolated hearts. The practical implications of our findings are that lactate did not rescue cardiac function and hemodynamics in ischemic HF. Instead, it contributed to decrease *mct1* mRNA expression and antioxidant enzymatic defense. Although increasing evidence suggests that switching metabolic fuel usage toward glycolytic fuel (e.g. lactate) oxidation increases cardiac function (8), our data provided evidence that it might not be helpful at all. In this sense, future studies should address the implication of the role of lactate in all phases of HF in cardiac gene expression and perfusion after MI.
